# Predictive Value of Corrected ^18^ F-FDG PET/CT Baseline Parameters for Primary DLBCL Prognosis: A Single-center Study

**DOI:** 10.1055/s-0044-1779282

**Published:** 2024-02-13

**Authors:** Min Li, Jianpeng Liu, Fangfei Liu, Rongbin Lv, Haowei Bai, Shuyong Liu

**Affiliations:** 1 Department of Nuclear Medicine, Tai'an Central Hospital of Qingdao University, Tai'an, Shandong, People's Republic of China; 2 Department of Radiology, Huashan Hospital of Fudan University, Shanghai, People's Republic of China; 3 Department of Nuclear Medicine, The Second Affiliated Hospital of Shandong First Medical University, Tai'an, Shandong, People's Republic of China

**Keywords:** ^18^ F-FDG PET/CT, DLBCL, progression-free survival, NCCN-IPI, prognosis

## Abstract

**Objective** The purpose of this study was to evaluate the prognostic significance of corrected baseline metabolic parameters in fluorodeoxyglucose positron emission tomography imaging ( ^18^ F-FDG PET/CT) for 3-year progression-free survival (PFS) in patients with primary diffuse large B cell lymphoma (DLBCL).

**Patients and Methods** Retrospective clinical and pathological data were collected for 199 patients of DLBCL diagnosed between January 2018 and January 2021. All patients underwent ^18^ F-FDG PET/CT scans without any form of treatment. The corrected maximum standardized uptake value (corSUVmax), corrected mean standardized uptake value (corSUVmean), corrected whole-body tumor metabolic volume sum (corMTVsum), and corrected total lesion glycolysis of whole body (corTLGtotal) were corrected using the SUVmean in a 1-cm diameter mediastinal blood pool (MBP) from the descending thoracic aorta of patients. Kaplan–Meier survival curves and Cox regression were used to examine the predictive significance of corrected baseline metabolic parameters on 3-year PFS of patients. The incremental values of corrected baseline metabolic parameters were evaluated by using Harrell's C-indices, receiver operating characteristic, and Decision Curve Analysis.

**Results** The multivariate analysis revealed that only the National Comprehensive Cancer Network (NCCN)-International Prognostic Index (IPI) and corMTVsum had an effect on 3-year PFS of patients ( *p* < 0.05, respectively). The Kaplan–Meier survival analysis demonstrated significant differences in PFS between the risk groups classified by corSUVsum, corMTVsum, and corTLGtotal (log-rank test, *p* < 0.05). The predictive model composed of corMTVsum and corTLGtotal surpasses the predictive performance of the model incorporating MTVsum and TLGtotal. The optimal performance was observed when corMTVsum was combined with NCCN-IPI, resulting in a Harrell's C index of 0.785 and area under the curve values of 0.863, 0.891, and 0.947 for the 1-, 2-, and 3-year PFS rates, respectively.

**Conclusion** The corMTVsum offers significant prognostic value for patients with DLBCL. Furthermore, the combination of corMTVsum with the NCCN-IPI can provide an accurate prediction of the prognosis.

## Introduction

Diffuse large B cell lymphoma (DLBCL) is the most prevalent form of non-Hodgkin lymphoma, constituting approximately 30 to 35% of cases. 1 2 It is a complex and heterogeneous malignancy characterized by a diffuse pattern of growth and aggressive behavior. While standard treatment with rituximab, cyclosphosphamide, doxorubicin, vincristine, and prednisone (R-CHOP) induces complete response in approximately 75 to 80% of patients diagnosed with DLBCL, a subset of approximately 10 to 15% of patients will present with primary refractory disease, and an additional 20 to 25% will experience relapse after initial response. 3

The International Prognostic Index (IPI) 4 and its modifications, age-adjusted IPI (aa-IPI), 5 and the National Comprehensive Cancer Network-IPI (NCCN-IPI), 6 have served as established prognostic models for DLBCL, helping clinicians make informed decisions and guiding treatment strategies for patients. While other prognostic factors, activated B cell-like subtype historically carries a less favorable prognosis 7 and protein expression patterns of MYC, BCL2 and BCL6, and MYC rearrangements were predictive of outcome and provided prognostic information independent of the IPI for overall survival (OS) and event-free survival. 8 However, most of the above indices were based on serum or fluorescence in situ hybridization, required a combination of several factors.

Fluorodeoxyglucose positron emission tomography imaging is a widely utilized technique for staging, restaging, and evaluating treatment response in DLBCL patients. 9 10 11 Various parameters that were measured during this process, such as maximum standard uptake value (SUVmax), metabolic tumor volume (MTV), and total lesion glycolysis (TLG), have demonstrated significant prognostic value in DLBCL. However, standardization of segmentation thresholds and measurement methods remains a crucial issue in the field of nuclear medicine literature, leading to inconclusive and contentious research outcomes with noticeable inconsistencies. 12 13 14 15

Accurate prognostic markers are essential for prognostic stratification, guiding timely interventions with novel treatments to improve their prognosis. Standardizing segmentation thresholds and measurement methods may also have significant impacts on clinical decision-making and ultimately improve patient outcomes with DLBCL. The principal objectives of our study were to evaluate the prognostic performance of corrected baseline tumor metabolic parameters and to determine the additional value in DLBCL patients, compared with the uncorrected baseline tumor metabolic parameters and NCCN-IPI.

## Materials and Methods

### Patients

Between January 2018 and January 2021, we retrospectively examined 199 patients diagnosed with DLBCL through lymph node or target lesion biopsy with postoperative pathological confirmation. All patients had complete clinical and follow-up data with a first-line R-CHOP chemotherapy regimen administered after the definitive diagnosis. Exclusion criteria included (1) a previous association with other malignancies, (2) progression or transformation from inert lymphoma, (3) prior malignancy-related treatment (including surgery, radiotherapy, and targeted drug therapy), (4) DLBCL of the central nervous system, (5) and comorbidities of acute and chronic active infectious diseases.

Follow-up information was obtained based on routine follow-up clinical examination, CT, and/or FDG-PET/CT scans. Progression-free survival (PFS) was defined as the duration from the initial diagnosis of the disease to the time of disease progression, recurrence, or death.

### Clinical/Histological Parameters

In this retrospective study, we evaluated the gender, age, erythrocyte sedimentation rate (ESR), hemoglobin level, number of extranodal invasion sites, lactate dehydrogenase (LDH), and ferritin levels of 199 patients diagnosed with DLBCL. Disease stages were classified according to the Ann Arbor staging criteria as I to IV, while patients were classified as having either A or B symptoms based on the presence or absence of unexplained fever of 38°C or higher, nocturnal sweating, and weight loss of 10% or more within 6 months. Physical status was evaluated using the Zubrod- Eastern Clinical Oncology Group (ECOG)-WHO (ZPS, 5-point scale) criterion, with scores ranging from 0 to 5. The IPI, aa-IPI, and the NCCN-IPI were calculated based on different criteria.

### Positron Emission Tomography Imaging Image Acquisition

Prior to undergoing an Ingenuity TF 128 PET/CT examination, patients were required to fast for 6 to 8 hours and maintain fasting blood glucose levels below 11.1 mmol/L. The imaging agent ^18^ F-FDG was injected via a superficial vein on the back of the hand at a dose of 3.7 MBq/kg, following which patients were instructed to rest calmly in the PET/CT lounge for approximately 50 to 60 minutes before the whole-body examination. Patients were advised to drink 500 mL of water and urinate prior to the exam. A whole-body PET/CT scan was conducted in the supine position, scanning from the cranial vault to the middle femur. This was followed by a 15- to 20-minutes PET scan while still in the same posture, followed by a CT scan with 128 rows at 120 kV, 100 to 150 mA, 0.75-mm pitch, and 3.75-mm layer thickness. PET data were corrected for attenuation and iteratively reconstructed using the self-contained software once the scan had been completed. Finally, all PET and CT images were transferred to a MEDEX computer workstation (Phillips Medical Systems), which constructs fused PET/CT images for further analysis.

### Positron Emission Tomography Imaging Image Analysis

To ensure accuracy, two diagnostic PET/CT physicians with more than 10 years of experience at the associate senior level or above collaborated to measure the SUVmean of systemic lesions as well as the SUVmean in the MBP of the descending thoracic aorta, which is 1 cm in diameter on PET/CT images. In the event of a disagreement, a consensus was achieved through consultation. The SUVmean of the lesion was corrected by the PET/CT diagnostician using the SUVmean of the patients' MBP of the descending thoracic aorta. The corSUVmean was calculated using the following formula: corSUVmean = patient whole-body lesion SUVmean × average MBP SUVmean/patients MBP SUVmean. The ratio of corSUVmean to SUVmean was used to determine corSUVmax. The region of interest (ROI) was determined using the threshold of 41% of corSUVmax proposed by the European Society of Nuclear Medicine. Corrected whole-body tumor metabolic volume sum (corMTVsum) was measured, and corrected total lesion glycolysis of whole body (corTLGtotal) = Σ (corSUVmean × corMTVsum). Lastly, based on the median, baseline metabolic parameters were separated into low- and high-risk groups.

### Statistical Analysis

This study conducted a statistical evaluation using R (version. 4.2.1). Continuous variables were presented as mean ± standard deviation or median with interquartile range (IQR), and categorical variables were reported as frequency and proportions. The R packages “survival” were used for Kaplan–Meier survival analyses and “rms” for Cox proportional hazards regression. Univariate regression analysis was conducted to obtain variables significantly related to PFS. In the uncorrected or corrected baseline parameters, significant variables identified through univariate regression analysis were used to construct the uncorrected and corrected model. All significant variables through univariate regression analysis were integrated into a multivariate regression analysis, then selected the significant variables to construct the combined models. Harrell's C-indices, ROC curve and Decision Curve Analysis (DCA) were used to compare the performance of the prediction. All tests were two-sided, with a significance level set at *p* -value less than 0.05.

## Results

### Patient Characteristics

**Fig. 1:**
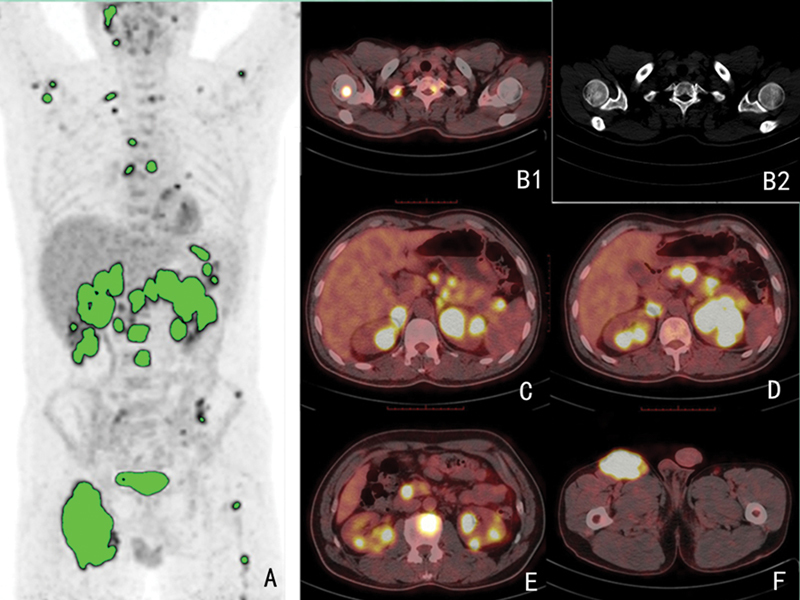
A 47-year-old male for primary DLBCL. Maximal intensity projection (MIP) image ( **A** ), bone window of CT ( **B2** ), and axial slice fused PET/CT ( **B1** , **C** – **F** ) show multiple enlarged lymph nodes in the body with markedly high FDG uptake. The corSUVmax, corSUVmean, corMTVsum, and corTLGtotal were 36.13, 21.48, 204.61, and 4262.18, respectively. corMTVsum, corrected whole-body tumor metabolic volume sum; corSUVmax, corrected maximum standardized uptake value; corSUVmean, corrected mean standardized uptake value; corTLGtotal, corrected total lesion glycolysis of whole body; DLBCL, diffuse large B cell lymphoma; FDG, fluorodeoxyglucose; PET/CT, positron emission tomography imaging.

The main characteristics of the patients are summarized in Table 1 . This study included a total of 199 patients with confirmed diagnosis of DLBCL, with a mean age of 62.91 ± 11.73 years and a range of 15 to 87 years. Complete follow-up information was obtained for all patients in the trial as of January 2021. Of the all patients, 91 patients experienced progression, relapse, or death, with a median time of 6.0 (IQR 4.0–9.0) months. In total of 108 patients did not experience such an event by the follow-up time, which had a median duration of 19.5 (IQR 13.0–27.0) months. A total of 25 patients died, with 3-year PFS and OS rates of 45.7 and 12.6%, respectively. Extranodal involvement was observed in 147 cases, with the gastrointestinal region being the most commonly affected (52 cases), followed by the bone (34 cases) the spleen (22 cases). A patient with multiple extranodal involvement is detailed in Fig. 1 .

**Table 1:** Baseline demographic and clinical characteristics of patients

Characteristics	*N* = 199 (%)
Sex	
Female	102 (51.3%)
Male	97 (48.7%)
Age (years)	
≥ 60	76 (38.2%)
> 60	123 (61.8%)
LDH	
Normal	98 (49.2%)
Elevated	101 (50.8%)
Hemoglobin	
Normal	99 (49.7%)
Elevated	100 (50.3%)
ESR	
Normal	64 (32.2%)
Elevated	135 (67.8%)
Ferritin	
Normal	97 (48.7%)
Elevated	102 (51.3%)
B symptoms	
No	105 (52.8%)
Yes	94 (47.2%)
ECOG PS	
0 to 1	141 (70.9%)
≥ 2	58 (29.1%)
ki-67	
< 80	74 (37.2%)
≥ 80	125 (62.8%)
Number of extralymph node invasions	
0 to 1	147 (73.9%)
≥ 2	52 (26.1%)
Ann Arbor stage	
I	36 (18.1%)
II	51 (25.6%)
III	48 (24.1%)
IV	64 (32.2%)
IPI	
0 to 1	54 (27.1%)
2	50 (25.1%)
3	26 (13.1%)
4 to 5	69 (34.7%)
aa-IPI	
0	30 (15.1%)
1	64 (32.2%)
2	36 (18.1%)
3	69 (34.7%)
NCCN-IPI	
0 to 1	16 (8.0%)
2 to 3	70 (35.2%)
4 to 5	68 (34.2%)
6 to 8	45 (22.6%)

Abbreviations: aa-IPI, age-adjusted International Prognostic Index; ECOG PS, Eastern Clinical Oncology Group Performance Status; ESR, erythrocyte sedimentation rate; IPI, International Prognostic Index; LDH, lactate dehydrogenase; NCCN-IPI, National Comprehensive Cancer Network-International Prognostic Index.

### Cox Regression Analysis

According to univariate regression analysis, sex, age, ESR, B symptoms, number of extralymph node invasions, SUVmax and SUVmean and corSUVmean were not the factors influencing patients 3-year PFS ( *p* > 0.05). Conversely, lactate dehydrogenase, hemoglobin, ferritin, Eastern Clinical Oncology Group Performance Status, Ann Arbor staging, IPI, aa-IPI, NCCN-IPI, MTVsum, and TLGtotal, corSUVmax, corMTVsum, and corTLGtotal were identified as significant factors that impacted PFS ( *p* < 0.05). Following the univariate regression analysis, only significant variables were subjected to further multivariate regression analysis. Based on the results, it was noted that NCCN-IPI (hazard ratio [HR] 3.077, 95% confidence interval [CI] 1.147–8.250) and corMTVsum (HR 7.099, 95% CI 2.642–19.078) were identified as the significant factors that influenced the 3-year PFS of patients, as shown in Table 2 .

**Table 2:** Univariable and multivariable Cox regression analyses

Potential factors	Univariable HR (95% CI)	Univariable *p*	Multivariable HR (95% CI)	Multivariable *p*
Sex				
Female ( *n* = 102)	Ref			
Male ( *n* = 97)	1.479 (0.978, 2.236)	0.064		
Age (years)				
≤ 60 ( *n* = 76)	Ref	–		
> 60 ( *n* = 123)	1.042 (0.681, 1.593)	0.851		
LDH				
Normal ( *n* = 98)	Ref	–	Ref	–
Elevated ( *n* = 101)	3.271 (2.072, 5.163)	< 0.001	1.014 (0.441–2.334)	0.973
Hemoglobin				
Normal ( *n* = 99)	Ref	–	Ref	–
Elevated ( *n* = 100)	2.875 (1.823, 4.533)	< 0.001	1.603 (0.941–2.732)	0.083
ESR				
Normal ( *n* = 64)	Ref	–		
Elevated ( *n* = 135)	1.112 (0.712, 1.735)	0.642		
Ferritin				
Normal ( *n* = 97)	Ref	–	Ref	–
Elevated ( *n* = 102)	1.913 (1.246, 2.935)	0.003	0.723 (0.427–1.225)	0.228
B symptoms				
No ( *n* = 105)	Ref	–	Ref	–
Yes ( *n* = 94)	1.155 (0.765, 1.743)	0.492		
ECOG PS				
0 to 1 ( *n* = 141)	Ref	–	Ref	–
≥ 2 ( *n* = 58)	1.646 (1.076, 2.520)	0.022	0.990 (0.596–1.643)	0.968
ki-67				
< 80 ( *n* = 74)	Ref	–	Ref	–
≥ 80 ( *n* = 125)	1.696 (1.075, 2.675)	0.023	1.099 (0.669, 1.808)	0.709
Number of extralymph node invasions				
to 1 ( *n* = 147)	Ref			
≥ 2 ( *n* = 52)	2.239 (1.470, 3.412)	0.642		
Ann Arbor Staging				
1 to 2 ( *n* = 87)	–		Ref	–
3 to 4 ( *n* = 112)	3.559 (2.164, 5.852)	< 0.001	1.143 (0.428–3.051)	0.790
IPI				
0 to 2 ( *n* = 104)	Ref	–	Ref	–
3 to 5 ( *n* = 95)	5.346 (3.293, 8.678)	< 0.001	2.509 (0.497–12.659)	0.265
aa-IPI				
to 2 ( *n* = 94)	Ref	–	Ref	–
3 to 5 ( *n* = 105)	4.965 (3.011, 8.187)	< 0.001	0.323 (0.053–1.949)	0.218
NCCN-IPI				
0 to 3 ( *n* = 86)	Ref	–	Ref	–
4 to 8 ( *n* = 113)	7.762 (4.215, 14.292)	< 0.001	3.077 (1.147–8.250)	0.026
SUVmax				
Low risk	Ref	–		
High risk	0.884 (0.585, 1.336)	0.559		
SUVmean				
Low risk	Ref	–		
High risk	0.715 (0.471, 1.084)	0.114		
MTVsum				
Low risk	Ref	–	Ref	–
High risk	7.092 (4.194, 11.993)	< 0.001	0.659 (0.252–1.724)	0.395
TLGtotal				
Low risk	Ref	–	Ref	–
High risk	4.611 (2.861, 7.433)	< 0.001	1.083 (0.504–2.326)	0.839
corSUVmax				
Low risk	Ref	–	Ref	–
High risk	1.539 (1.014, 2.336)	0.043	1.029 (0.606–1.559)	0.905
corSUVmean				
Low risk	Ref	–		
High risk	1.153 (0.764, 1.740)	0.498		
corMTVsum				
Low risk	Ref	–	Ref	–
High risk	11.807 (6.485, 21.494)	< 0.001	7.099 (2.642–19.078)	< 0.001
corTLGtotal				
Low risk	Ref	–	Ref	–
High risk	7.274 (4.266, 12.402)	< 0.001	1.588 (0.592–4.261)	0.358

Abbreviations: aa-IPI, age-adjusted International Prognostic Index; corMTVsum, corrected whole-body tumor metabolic volume sum; corSUVmax, corrected maximum standardized uptake value; corSUVmean, corrected mean standardised uptake value; corTLGtotal, corrected total lesion glycolysis of total body; ECOG PS, Eastern Clinical Oncology Group Performance Status; ESR, erythrocyte sedimentation rate; IPI, International Prognostic Index; LDH, lactate dehydrogenase; NCCN-IPI, National Comprehensive Cancer Network-International Prognostic Index.

### Survival Analysis

**Fig. 2:**
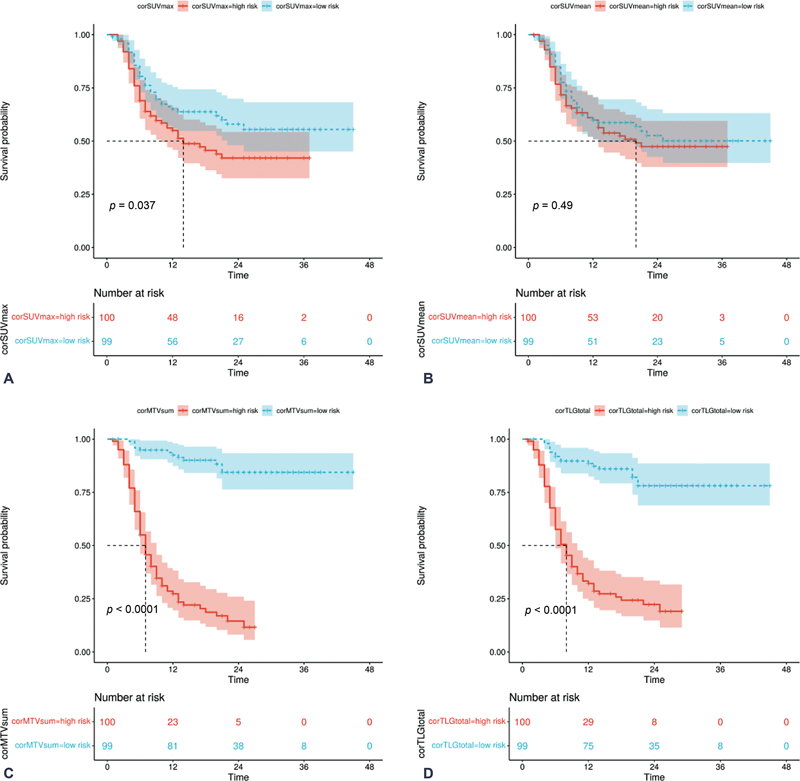
Kaplan–Meier curves evaluating progression-free survival using corrected baseline tumor metabolic parameters, including corSUVmax ( **A** ), corSUVmean ( **B** ), corMTVsum ( **C** ), and corTLGtotal ( **D** ). corMTVsum, corrected whole-body tumor metabolic volume sum; corSUVmax, corrected maximum standardized uptake value; corSUVmean, corrected mean standardized uptake value; corTLGtotal, corrected total lesion glycolysis of whole body.

Kaplan–Meier estimates showed that patients could be stratified into low- or high-risk according to median of corrected baseline tumor parameters. The median SUVmax, SUVmean, MTVsum, and TLGtotal was 22.610 (IQR 16.150, 28.830), 12.802 (IQR 9.100, 17.011), 60.282 (IQR 15.832, 180.543), and 642.247 (IQR 215.270, 2807.637), respectively. The median corSUVmax, corSUVmean, corMTVsum, and corTLGtotal was 19.550 (IQR 13.878, 27.718), 10.924 (IQR 7.669, 15.348), 49.783 (IQR 13.410, 173.735), and 678.474 (IQR 161.732, 2667.590), respectively. A statistically significant difference (log-rank test, *p* < 0.05) in PFS between the risk groups categorized by corSUVmax, corMTVsum, and corTLGtotal. However, no significant differences were observed between the risk groups categorized by corSUVmean (log-rank test, *p* > 0.05). These results are depicted in Fig. 2A–D .

### Performance of the Prediction

**Fig. 3:**
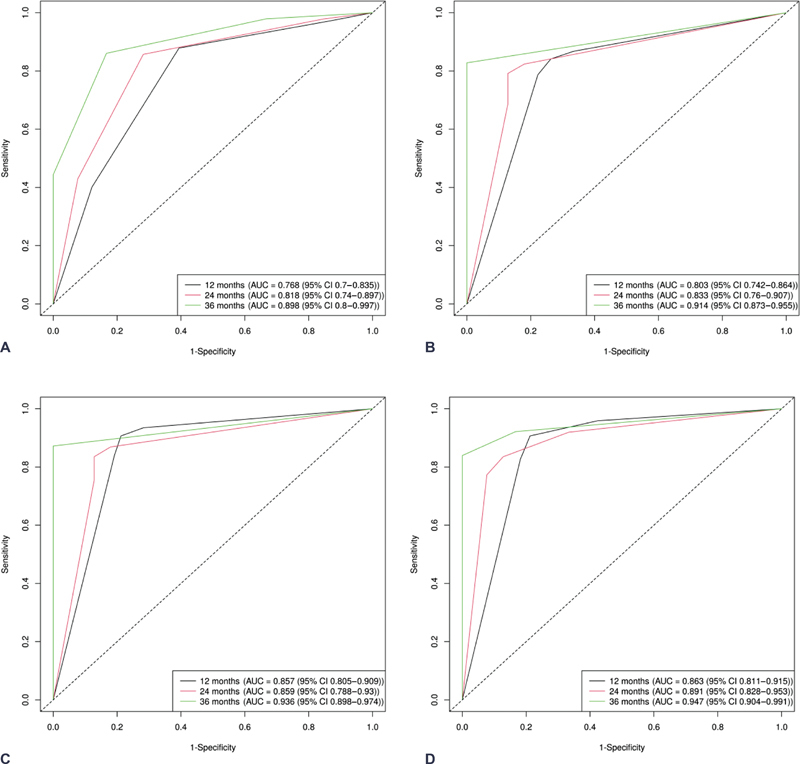
ROC analysis curve for prediction of 1-, 2-, 3-year PFS in NCCN-IPI ( **A** ) MTVsum and TLGtotal ( **B** ), corMTVsum and corTLGtotal ( **C** ), and corMTVsum and NCCN-IPI ( **D** ). CI, confidence interval; corMTVsum, corrected whole-body tumor metabolic volume sum; corTLGtotal, corrected total lesion glycolysis of whole body; NCCN-IPI, National Comprehensive Cancer Network-International Prognostic Index; PFS, progression-free survival; ROC, receiver operating characteristic.

**Fig. 4:**
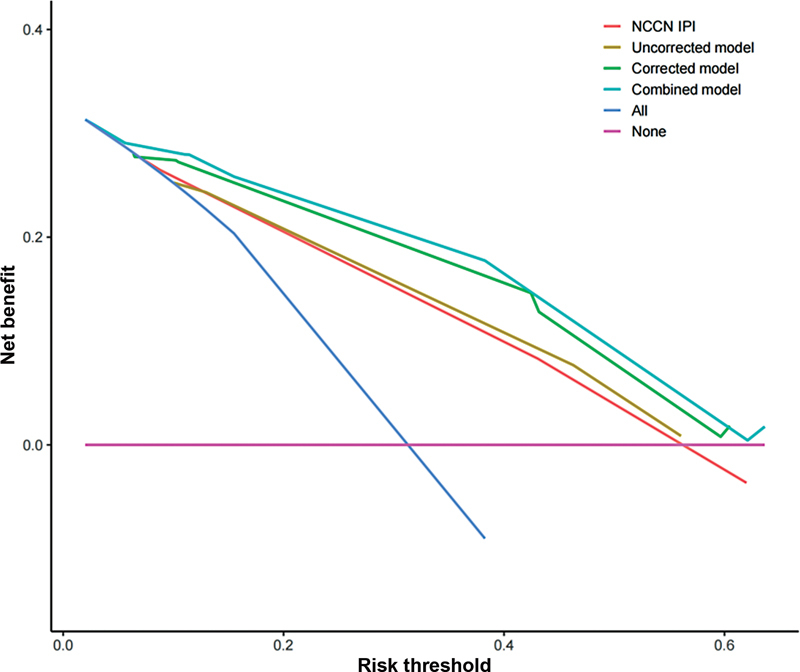
Decision curve analysis for the NCCN-IPI, uncorrected model, corrected model, and combined prediction model in the progression-free survival. Net benefit was calculated as follows: Net benefit = true positive rate − (false positive rate × weighting factor). Weighting factor = threshold probability/1 − threshold probability. NCCN-IPI, National Comprehensive Cancer Network-International Prognostic Index.

The NCCN-IPI, MTVsum and TLGtotal, corMTVsum and corTLGtotal all showed predictive ability for PFS, with C-indices of 0.730 (95% CI 0.684, 0.776), 0.731 (95% CI 0.685, 0.778), and 0.769 (95% CI 0.728, 0.811), respectively. Notably, the combined model, incorporating NCCN-IPI and corMTVsum, outperformed above predictive model, achieving a C-index of 0.785 (95% CI 0.746, 0.824) for predicting PFS. The predictive model composed of corMTVsum and corTLGtotal (area under the curve [AUC] 0.857, 0.859, and 0.936 for 1-, 2-, and 3-year PFS) surpasses the predictive performance of the model incorporating MTVsum and TLGtotal (AUC 0.803, 0.833, and 0.914 for 1-, 2-, and 3-year PFS). The combined model derived from a multivariate regression analysis, incorporating NCCN-IPI and corMTVsum, attains the highest AUC (AUC 0.863, 0.891, and 0.947 for 1-, 2-, and 3-year PFS). Notably, all these models above superior performance compared with NCCN-IPI (AUC 0.768, 0.818, and 0.898 for 1-, 2-, and 3-year PFS). Detailed results are provided in Fig. 3 and Table 3 . DCA demonstrated a notably higher net benefit for both the combined and corrected models compared with the uncorrected model and NCCN-IPI across diverse threshold probabilities in predicting PFS ( Fig. 4 ).

**Table 3:** Receiver operating characteristic analysis curve for prediction of 1-, 2-, 3-year progression-free survival in prediction models

	1-year PFS		2-year PFS		3-year PFS	
	AUC	95% CI	AUC	95% CI	AUC	95% CI
NCCN-IPI	0.768	(0.700, 0.835)	0.818	(0.740, 0.897)	0.898	(0.800, 0.997)
MTVsum and TLGtotal	0.803	(0.742, 0.864)	0.833	(0.760, 0.907)	0.914	(0.873, 0.955)
corMTVsum and corTLGtotal	0.857	(0.805, 0.909)	0.859	(0.788, 0.930)	0.936	(0.898, 0.974)
Combined model	0.863	(0.811, 0.915)	0.891	(0.828, 0.953)	0.947	(0.904, 0.991)

Abbreviations: AUC, area under the curve; CI, confidence interval; corMTVsum, corrected whole-body tumor metabolic volume sum; corTLGtotal, corrected total lesion glycolysis of total body; NCCN-IPI, National Comprehensive Cancer Network-International Prognostic Index; PFS, progression-free survival.

## Discussion

The MTV and TLG volumetric parameters have been recognized as reliable indicators for survival prediction in DLBCL patients. However, the lack of a standardized methodology for calculating these values hinders their broader clinical application. To improve accuracy and provide more meaningful results in practice, it is crucial to develop a normalized methodology for calculating these parameters. In this study, we have corrected baseline metabolic parameters to take into account the radiochemical purity and dose of each patient. Our study showed that baseline corSUVsum, corMTVsum, and corTLGtotal were significantly correlated with DLBCL. Improved prediction accuracy (Harrell's C rose from 0.731 to 0.769) for PFS was achieved by incorporating corMTVsum and corTLGtotal over MTVsum and TLGtotal. It also revealed that the combined model incorporating corMTVsum and NCCN-IPI provided superior predictive performance (AUC: 0.863, 0.891, and 0.947 for 1-, 2-, and 3-year PFS). These results outperformed the NCCN-IPI model and highlight the potential of our methodology to predict prognostic accuracy in clinical practice for patients diagnosed with DLBCL.

MTV has been regarded as a potential biomarker for the development of initial PET-adapted approaches in follicular lymphoma. 16 However, debates have arisen regarding the predictive value of baseline metabolic parameters in patients with DLBCL and the existing studies have presented conflicting results. Shagera et al 17 discovered that prechemotherapy MTV was an independent prognostic factor in addition to NCCN-IPI. The combination of baseline TMTV and NCCN-IPI may have the potential to improve the prognosis of DLBCL patients with high-risk NCCN-IPI group. On the other hand, Sasanelli et al 18 found that MTV was the only independent factor in the prognosis of DLBCL patients, while TLG was not significant. Zhou et al 15 reported that baseline TLG was an independent predictor of survival in DLBCL patients before R-CHOP treatment. Nevertheless, diverging from these results, Gallicchio et al 19 revealed that only SUVmax was a predictor of PFS in DLBCL patients, and lower SUVmax values were associated with worse outcomes.

To ensure the reliability and comparability of PET imaging parameters across centers, we propose a collaborative effort similar to the Image Biomarker Standardization Initiative. This study utilizes a hybrid measurement method that combines the fixed threshold of corSUVmax method with the threshold of 41% corSUVmax method to calculate baseline parameters. The determination of the fixed threshold for corSUVmax is based on the average SUVmean in the MBP form the Deauville criteria. It is a 5-point scale model, implemented to standardize the visual assessment of treatment response in DLBCL through interim and post-treatment FDG-PET/CT. 20 In this scale model, the FDG uptake in the MBP and liver is referenced. 21 Barrington et al 22 delineated the methodology and location for measuring SUV in reference regions, but there remains a lack of uniformity in the measurement details. Variations include the choice between SUVmean and SUVmax, as well as the use of ROI versus volume of interest. To enhance reproducibility, we adopted a 1-cm diameter ROI within the MBP from the descending thoracic aorta to correct the SUVmean. In contrast, the SUVmean of the liver was not used to normalize the SUVmean of the whole-body lesions since many patients had liver involvement, and the SUVmean of the affected liver was substantially higher than the normal liver. Additional correction methods, such as partial volume correction (PVC), have demonstrated enhanced accuracy in quantifying PET images in cancer studies. 23 24 A previous investigation, the application of PVC improved the accuracy of TLG for predicting survival in patients with relapsing/refractory lymphoma before and after radioimmunotherapy. 25 Our study utilized the SUVmean of the MBP to eliminate the heterogeneity caused due to unequal radioactivity during the FDG injection or varying doses of patients owing to their body weight or other factors. After correction, the corSUVmax emerged as a predictive variable for PFS in univariate regression analysis than SUVmax without correction. With a 100% follow-up rate, cor SUVmax, corMTVsum, and corTLGtotal were all significantly correlated with PFS in DLBCL before R-CHOP treatment.

To evaluate 3-year PFS in patients with primary DLBCL, we combined all available clinical variables that could impact prognosis with PET/CT baseline metabolic parameters. B symptoms, 26 anemia, 27 elevated levels of ESR 28 and ferritin 29 have been proved to be poor prognostic factors in DLBCL patients. However, conflicting results have been reported regarding their impact on prognosis. Our study confirms that anemia and elevated levels of ferritin are associated with a poor prognosis, but we did not observe B symptoms and ESR levels as significant predictors of outcomes.

Our study has several limitations that should be noted. This study was a single-center retrospective study, a small sample size and heterogeneous population may have introduced potential bias and confounding variables that could have influenced the identified clinical prognostic factors. Additionally, we did not perform molecular pathology analysis, including assessments of MYC, BCL-2, and BCL-6 protein expression and gene rearrangements, known as prognostic factors. This limitation arose from the unavailability of data for all patients. To further explore the potential of combining baseline tumor metabolic characteristics and radiomics for personalized prognosis in DLBCL patients and to assess whether it could eventually replace the NCCN-IPI, future prospective research is warranted.

## Conclusion

Corrected PET-CT baseline metabolic parameters can serve as critical prognostic factors for primary DLBCL patients. A combined model incorporating corMTVsum with the NCCN-IPI can provide additional prognostic value to PFS in primary DLBCL patients. Ultimately, this approach may lead to the development of more precise and individualized treatment plans, thereby enhancing the accuracy of prognostication and improving patient outcomes in primary DLBCL patients.
